# Reports on the Hospitals of the United Kingdom

**Published:** 1909-11-27

**Authors:** Henry Burdett


### REPORTS
ON THE
### Hospitals of the United Kingdom.

By SIR HENRY BURDETT, K.C.B., K.C.V.O.

### XI.—SOUTH DEVON AND EAST CORNWALL HOSPITAL, PLYMOUTH.

This institution was established in 1840. It was rebuilt about 22 years ago. The buildings on the whole present a very charming picture, and the pavilions of two stories are so arranged and designed that they collectively group themselves together in the most attractive harmony. The reason for the beauty of the buildings as a whole is that they are built of greystone, which suits the climate and atmosphere of Plymouth, and gives them a beauty and strength probably unique in this type of building.

### Notable Defects.

The hospital is entered by a gate at one corner, and the puzzle is for the stranger to make out which block constitutes the administration building. The entrance to this block is mean, and consists of a long corridor with rooms on each side, which make the great defect of the general design apparent at once. There is no reception room, nor any accommodation of any kind provided where visitors, either on business or the friends of patients, can wait. A wooden bench in the main corridor is the only hospitality extended to the visitor, and the consequence is that the corridor is necessarily untidy and sometimes overcrowded. Again, it is of the first importance to an institution supported by voluntary contributions that each visitor should be favourably impressed upon entering the hospital. At Plymouth this important feature of hospital administration is necessarily absent, and the injury to the general reputation of the hospital may be, and often is, increased by the presence of patients waiting to see a member of the staff, of patients' friends, and of visitors on business, who have to stand about together in the somewhat narrow and dark corridor of entrance. Anyone who refers to the report and sees the financial condition of the hospital must be surprised that the committee tolerate this growing defect for a single moment. They have, as we shall proceed to show presently, ample funds at their disposal to defray the cost of the necessary reconstruction.

Another important improvement, which is urgently called for and could be readily carried out, is the enlargement of the verandahs at the end of each ward, from which magnificent views are obtainable, so as to enable patients to be wheeled out in their beds and to have the full benefit of complete open-air treatment at all seasons of the year. Indeed, there would appear to be some need for open-air treatment at this hospital, as the report records that the average stay of each in-patient during the year is from 36 to 39 days. This is a remarkable fact in these days of aseptic treatment, especially when the excellence of the site and the quality of the air of Plymouth are considered. Nowadays in the best-administered hospitals the average stay of surgical cases, and, indeed, of all acute cases, seldom exceeds 19 days. We should like to have a full explanation of the reasons which have brought about this excessive residence of each in-patient at the South Devon and East Cornwall Hospital.

Another point where we think the authorities make a mistake like those at the Exeter Infirmary is that they do not keep the internal decoration and appearance of the hospital sufficiently smart and attractive to meet modern requirements and secure the increasing and generous support of those who take an intelligent interest in a well-managed hospital.

### Wanted, a Nursing Home.

This institution, though it has a private nursing staff, does not possess a nurses' home. It owns a few houses which are devoted to the accommodation of the nurses, and possesses the novelty of one entire house devoted to the night nurses! Having regard to the importance of this hospital as the chief institution of the kind for the inhabitants of the unusually large area which it serves, it is essential that it should possess without further delay a large, well-planned, up-to-date nurses' home, with its own separate kitchen, so that the nursing staff may be economically and well fed. The fact of such economy can only be shown by the publication of separate accounts of the nursing department under all heads in the income and expenditure accounts each year.

We can well understand that the average supporter of this hospital, on reading the foregoing suggestions and criticisms, will exclaim: "Give us the money, and we will carry out the reconstructions and reforms suggested. We have no funds available at present, and therefore can do nothing." An examination of the accounts shows that although the excess of expenditure over income for the year 1908 amounted to £1,931, as shown on the face of the accounts, a sum of £2,162 was received from legacies, so that, in fact, the total revenue exceeded the total expenditure by upwards of £200. The same may be said of previous years, so that the outstanding deficiency of the past and previous years, amounting as it does to £3,496, is in fact but a book entry. We are aware that certain of the legacies were left for investment, but the members of the Finance Committee, as men of business, ought to recognise that investments amounting to £103,549 is a sum out of all proportion to the needs of a hospital so far as security is concerned, whereas in the present case, the total expenditure is less than £10,000 per annum. If the invested funds amounted to £50,000, they would be ample for a hospital like the South Devon and East Cornwall with its present annual expenditure. We should therefore counsel the governors to take steps to have plans and estimates prepared for carrying out the reconstruction scheme we have ventured to indicate, and to take the money from the present invested property account.

### How to Increase the Income.

The Committee, in their report, point out that the ordinary expenditure exceeds the ordinary income on an average by some £1,750. We are surprised that the excess is not larger than these figures show, for we find that annual subscribers of two guineas under the present scale of privileges can send in one in-patient each year, whilst the average cost of each in-patient is in round figures £7. That is to say, every subscriber of two guineas, as matters stand at present, who uses his in-patient letter, receives back from the hospital in actual value seven pounds for each two guineas paid to the hospital. This is not charity, or philanthropy, or business, and the sooner a committee is appointed to revise the present scale of privileges for subscribers, and to put them on a more equitable footing, the better for everybody concerned. Many hospitals have had to adopt this course, with the result that they have speedily adjusted the income account and made it ample to defray the whole ordinary expenditure of each year, whilst the popularity and standing of the hospital has been really strengthened in consequence.

### Errors to be Avoided.

Finally, we were surprised to find that, owing to the request, as we were informed, of some members of the medical staff, the new medical ward block has been spoilt by the inadequate number of windows placed in each ward, whereby necessary light and air have been excluded. These new wards represent distinct retrogression in hospital construction. For this reason they are well worth a visit from intelligent hospital managers who desire to have a practical lesson in what to avoid. In any case, these wards must go down to history as a remarkable instance of how mistakes and errors in hospital construction are sometimes brought about by hospital authorities, owing to their failure to take the ordinary safeguards which the advice of an authoritative and experienced referee can bring to bear on all matters of doubt and difficulty whenever they arise.

---

The **Crumpsall Workhouse Infirmary, Manchester** will be reported on next week.

